# Enhanced antifungal activity of bovine lactoferrin-producing probiotic *Lactobacillus casei* in the murine model of vulvovaginal candidiasis

**DOI:** 10.1186/s12866-018-1370-x

**Published:** 2019-01-08

**Authors:** Hong Liao, Shanling Liu, He Wang, Hang Su, Zhenjun Liu

**Affiliations:** 1 Department of Obstetric & Gynecologic, West China Second University Hospital, Sichuan University, NO. 17.3rd Section, Renmin South Road, Chengdu, Sichuan 610041 China; 2 Key Laboratory of Birth Defects and Related Diseases of Women and Children (Sichuan University), Ministry of Education, Chengdu, China

**Keywords:** Vulvovaginal candidiasis, Bovine lactoferrin, Antimicrobial peptides, *Lactobacillus casei*, Antifungal activity, Heterologous expression

## Abstract

**Background:**

Vulvovaginal candidiasis (VVC) is a common vaginitis caused by *Candida* species,a frequently recurring condition. Fungal azole-resistant strains with azole-resistance have developed for long and wide explosion to the first-line antifungal azole agent. Bovine lactoferrin (BLF) is a protein from transferrin family secreted by the bovine mammary tissue. Its various biological functions are well known, especially the pronounced antifungal activity.

**Results:**

In the current study, we constructed a *Lactobacillus casei* strain (*L.casei*/pPG612.1-BLF), which secreted BLF encoded by a mature secretion vector plasmid pPG612.1, and evaluated its antifungal activity in vitro and in vivo. In a two-layer agar plate in vitro assay, the number of *C. albicans* CFUs decreased and the average colony size shrunk upon exposure to *L. casei*/pPG612.1-BLF. In a murine VVC model, the infection burden of mice intra-vaginally pre-inoculated with *L. casei*/pPG612.1-BLF was lower than in control groups. Furthermore, the infection burden in mice with VVC was reduced when the animals were continually given *L. casei*/pPG612.1-BLF as a topical treatment for 5 days.

**Conclusion:**

Combined, these results suggested that the *L. casei*/pPG612.1-BLF strain is a promising preventative and therapeutic anti-VVC agent, highlighting the possibility of employing the probiotic *L. casei* as a vehicle for biotherapy in the female genital tract and exploiting the natural antibiotic antimicrobial peptides for other applications.

## Background

Vulvovaginal candidiasis (VVC) is the second most common infection disease in female genitourinary tract after bacterial vaginosis. It can affect up to 75% of women of childbearing age [1]. Symptoms, including burning pain, intense pruritus and abnormal “cheese-like” or watery vaginal discharge, are typical [2, 3]. *Candida. Albicans* (*C. albicans*), identified as the most common pathogen in VVC, is commensal fungi colonizing in female genitourinary tracts [4]. Certain risk factors, e.g., oral contraceptive usage, pregnancy, uncontrolled diabetes mellitus, and long-term broad spectrum antibiotic treatment, may cause it to become pathogenic [5–7]. The virulence factors were considered as the morphogenetic transition, the expression of adhesins and invasins, the formation of biofilms, phenotypic switching and the secretion of hydrolytic enzymes when invading the host [8].

Clinically, azoles are the first-line agents both in topical and oral VVC treatment [9, 10]; however, fungal strains exhibiting azole resistance, especially fluconazole resistance, have emerged [11, 12]. Research on new therapeutic agents, e.g., azole derivatives and amphotericin B, nikkomycin Z, and antimicrobial peptides (AMPs), shows their promising fungicidal activity; otherwise, preventative approaches, e.g., vaccines interfering with immunological sensitization, are also feasible [13–15].

AMPs are widely recognized as a group of cationic peptides with antimicrobial and immuno-modulating properties; they are produced by a variety of species [16, 17]. As key components of the innate immunity, they function as the first barrier defending the host against a wide array of pathogens, from bacteria and fungi to protozoa [18]. Bovine lactoferrin (BLF), a member of the transferrin family of proteins with various biological functions, is abundant in bovine milk glycoproteins as well as in other secreted fluids [19]. It has attracted a lot of attention because of its pronounced bactericidal and fungicidal activity against a wide range of microorganisms at the mucosal surfaces [20, 21]. As a natural AMP agent with little toxicity to the host cells, BLF is a subject of research for its potential use in treatment and prevention of fungal infections [22, 23].

*Lactobacillus* species, Gram-positive bacteria, are dominant members of the natural microbiota of the vaginal and gastrointestinal tract, maintaining the vaginal microenvironment to prevent pathogen invasion [24, 25]. Since they are regarded as safe, *Lactobacillus* strains are widely employed by the fermented food and beverage industry, and used as vaccine delivery systems to activate the mucosal immunity in animal models [26, 27]. Some studies demonstrated the effectiveness of topical or oral administration of certain *Lactobacillus* strains as probiotics in preventing the recurrence of VVC, but consistent evidence of their effectiveness still lacking [28, 29]. Further, no investigations concerning the utilization of AMP-producing *Lactobacillus* strains in the vaginal tract have been reported. In the current study, we constructed a BLF-producing system based on the plasmid pPG612.1 and *Lactobacillus casei ATCC 393*, and evaluated its preventative and therapeutic activity in the murine model of VVC.

## Results

### Construction of a *L. casei* strain secreting BLF

**Fig. 1:**
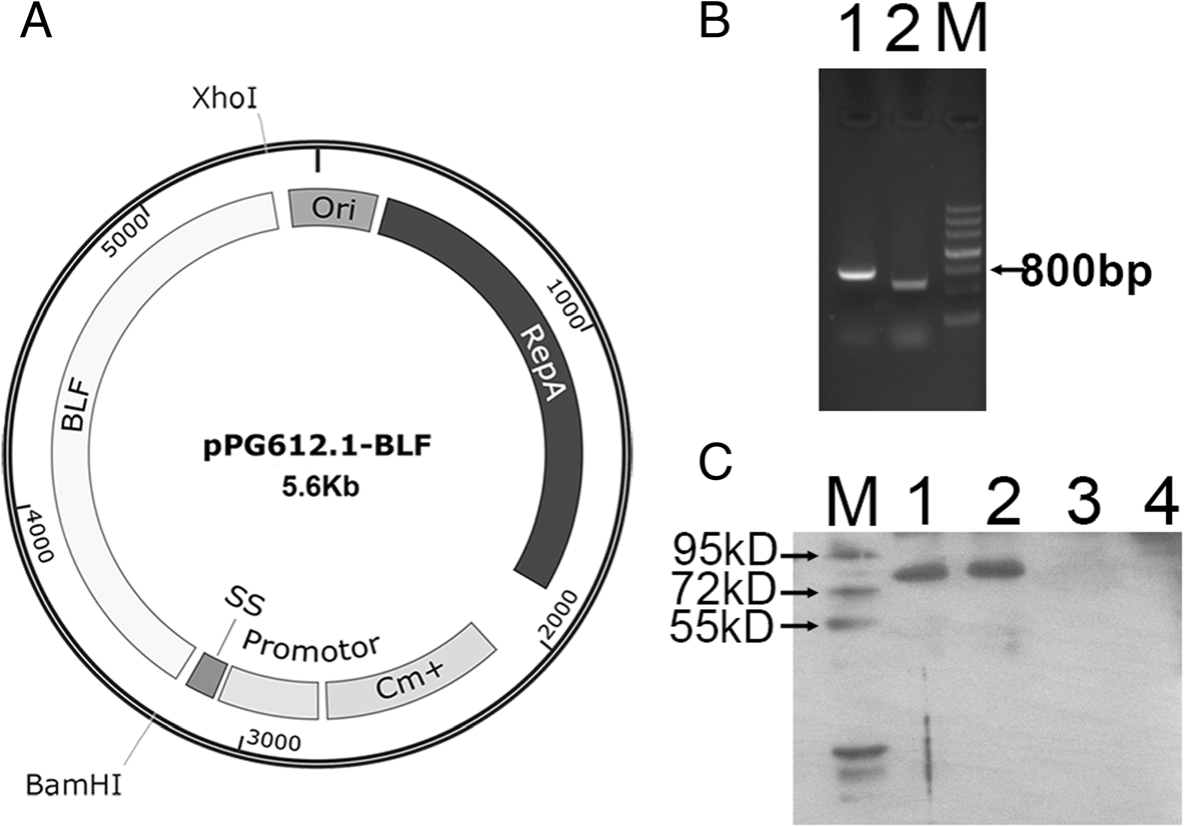
Construction and expression of the secretion plasmid pPG612.1-BLF in *L.casei*. **a** The synthetic BLF gene fragment (2.1kp) was digested with restriction enzymes BamHI and XhoI, and ligated into the sticky end of the plasmid pPG612.1 which was also digested with the same restriction enzyme, resulting in the plasmid pPG612.1-BLF (5.6kp). **b** The plasmid pPG612.1-BLF was electroporated into *L.casei* using a BioRad GenePulser with single electric pulse (voltage, 1.5 kV; capacitance, 25 μF; and resistance, 400 Ω.). PCR amplification of the BamHI site and XhoI site of the plasmid pPG612.1-BLF which was extracted from the *L.casei*/pPG612.1-BLF strain resulted in 500 bp and 800 bp products, respectively. Lane 1, PCR product of XhoI site; Lane 2, PCR product of BamHI site. M, DNA maker. **c** BLF was detected in the supernatant and pellet of *L.casei*/pPG612.1-BLF culture by Western blotting, indicating the expression and secretion of BLF by *L.casei*/pPG612.1-BLF. Lane 1, supernatant of *L.casei*/pPG612.1-BLF culture; Lane 2, pellet of *L.casei*/pPG612.1-BLF culture; Lane 3, supernatant of *L.casei*/pPG612.1 culture; Lane 4, pellet of *L.casei*/pPG612.1 culture

The *L. casei*/pPG612.1-BLF harboring the plasmid pPG612.1-BLF was grown on MRS agar or broth supplemented with 10 μg/mL Cm. Amplification of the BamHI and XhoI sites of the plasmid pPG612.1-BLF isolated from the *L. casei*/pPG612.1-BLF strain resulted in 500-bp product; sequencing of the PCR products verified the successful insertion of the BLF coding sequence in plasmid pPG612.1(data not shown). Following protein precipitation, electrophoresis, and western blotting, Immunoreactive bands corresponding to BLF (78 kD) were observed between the 72-kD and 95-kD size markers on film, both in the supernatant and pellet of the *L. casei*/pPG612.1-BLF overnight culture, but not in untransformed *L. casei* cells (Fig. 1).

### Fungicidal effects of *L. casei*/pPG612.1-BLF against C.albicans in vitro

**Fig. 2:**
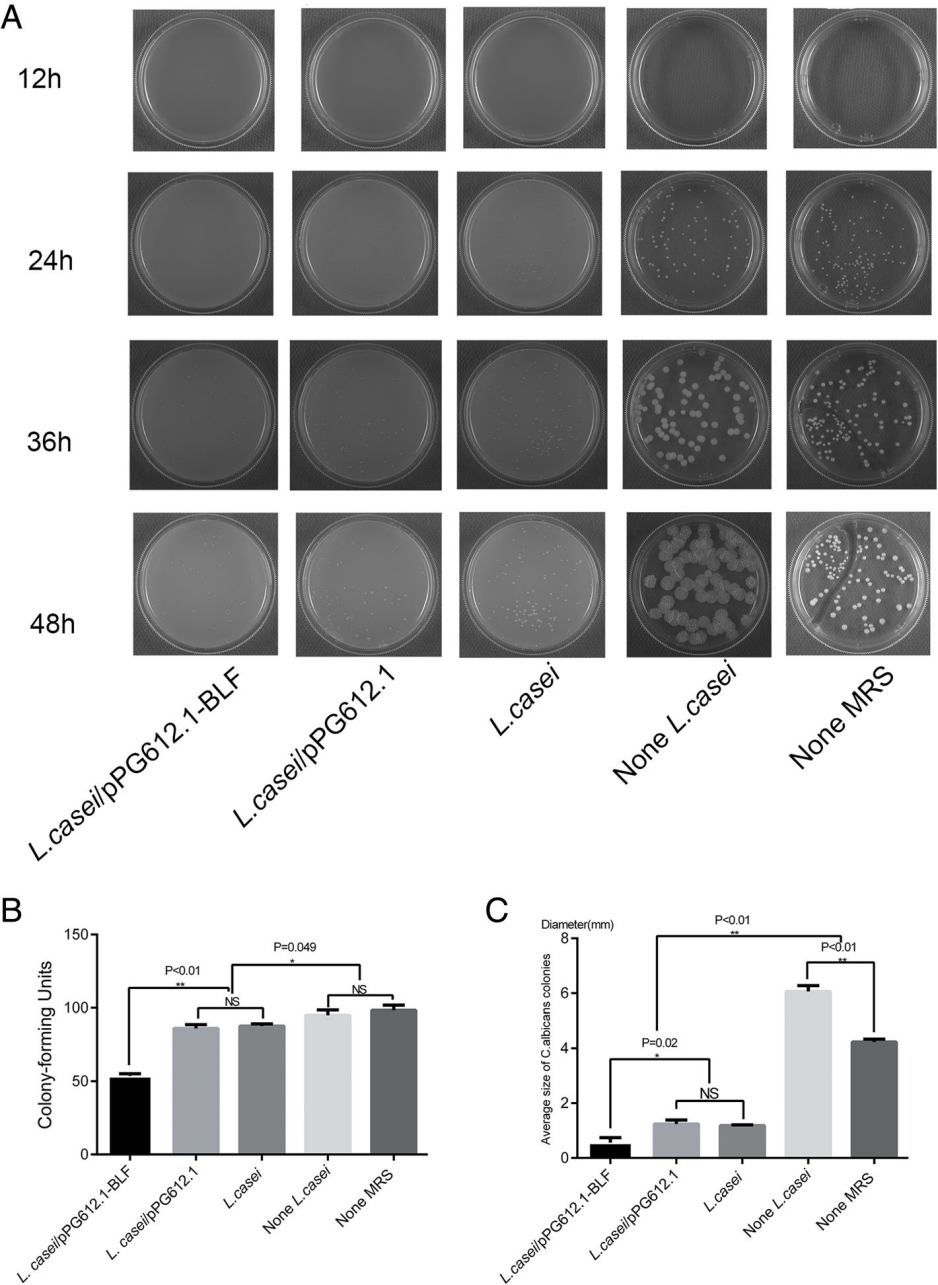
Antifungal activity of *L.casei*/pPG612.1-BLF in vitro. **a** C.albicans and *L.casei*/pPG612.1-BLF were incubated together on a two-layer agar dish at 37 °C for 48 h, *C.albicans* on the top SDA layer and L.casei/pPG612.1-BLF in the bottom MRS layer. Photos were taken every 12 h. **b** Twenty microliter of L.casei/pPG612.1-BLF (OD600 = 0.6, 5 × 10^7^–1 × 10^8^ cells/mL) was added into 200 ml of MRS medium with1.5% low melting agarose and mixed gently in the 37 °C water bath. Before cooling down, 20 ml of the mix was poured onto each culture dish (60 mm diameter) and allowed to solidify. Another 10 ml of SDA with 1.5% low melting agarose was poured on to the top of the MRS agar, and then allowed to solidify. Following that, 100 μL of *C.albicans* liquid containing about 100 cells was placed on the surface of the two-layer agar dish. Two-layer agar dishes containing *L.casei*/pPG612.1, *L.casei* and none *L.casei* were taken as negative controls, one-layer SDA dishes containing *C.albicans* alone (None MRS) were taken as blank controls. The CFUs of C.albicans incubated with *L.casei*/pPG612.1-BLF (53.67 ± 1.53CFUs) were fewer than that grown with *L.casei*/pPG612.1 (86.00 ± 2.64CFUs) and L.casei (87.67 ± 1.53CFUs) (*P* < 0.01), but there was no difference of CFUs between the latter two groups. Additionally, the CFUs of C.albicans without *L.casei* (None *L.casei*, 95.00 ± 3.61CFUs) and without MRS (None MRS, 98.33 ± 3.51 CFU) were more than those with *L.casei*/pPG612.1 or *L.casei*. (C) The average size of C.albicans CFUs with *L.casei*/pPG612.1-BLF (0.57 ± 0.18 mm) was smaller than that with *L.casei*/pPG612.1 (1.23 ± 0.15 mm) and *L.casei* (1.18 ± 0.03 mm) (*P* = 0.02). *C.albicans* CFUs on two-layer dishes without *L.casei* (None *L.casei,* 6.07 ± 0.21 mm) was flatter and bigger than that grown without MRS dishes (None MRS, 4.23 ± 0.10 mm) (*P* < 0.01)

We developed a two-layer agar dish assay, which allowed *C. albicans* to be directly exposed to the secretions of the *L. casei*/pPG612.1-BLF; i.e., *L. casei* cells were grown anaerobically in the bottom MRS layer, while *C. albicans* cells were grown on the surface of the top SDA layer. The quantity and average size of *C. albicans* colonies were analyzed to evaluate the antifungal effect of BLF produced by *L. casei*/pPG612.1-BLF. After a 24-h incubation, *C. albicans* colonies formed in the absence of *L. casei* were readily discernible, but they were smaller after incubation in the presence of *L. casei*/pPG612.1-BLF and *L. casei* (Fig. 2a). After 48 h of incubation, the colonies in each group were counted, and the colony diameter was measured using a vernier caliper. As observed, the CFU number of *C. albicans* grown in the presence of *L. casei*/pPG612.1-BLF (53.67 ± 1.53) was lower than of cells grown with *L. casei*/ pPG612.1 (86.00 ± 2.64) or *L. casei* (87.67 ± 1.53) (*P* < 0.01); no difference in CFU numbers was apparent between the two latter groups (Fig. 2b). Additionally, the CFU numbers of *C. albicans* grown in the absence of *L. casei* (95.00 ± 3.61) and without MRS (98.33 ± 3.51) were higher than those of cells grown in the presence of *L. casei*/pPG612.1 or *L. casei*.

Regarding the average colony size in each group, the fungal colonies grown in the presence of *L. casei*/pPG612.1-BLF (0.57 ± 0.18 mm) were smaller than those of cells grown in the presence of *L. casei*/pPG612.1 (1.23 ± 0.15 mm) or *L. casei* (1.18 ± 0.03 mm) (*P* = 0.02); no difference was apparent between the two latter groups (Fig. 2c). After 36 h of incubation, *C. albicans* colonies that grew on the two-layer plates in the absence of *L. casei* (6.07 ± 0.21 mm) appeared to be flatter and bigger than those grown on SDA without MRS (4.23 ± 0.10 mm) (*P* < 0.05), indicating that MRS medium alone could contribute to the growth of *C. albicans.* Overall, these results suggested that BLF secreted by the *L. casei*/pPG612.1-BLF strain infiltrated the SDA, interfering with the growth of *C. albicans* ATCC 10231, as reflected by the colony number and average colony size.

### Protective effect of *L. casei*/pPG612.1-BLF in the VVC mouse model

**Fig. 3:**
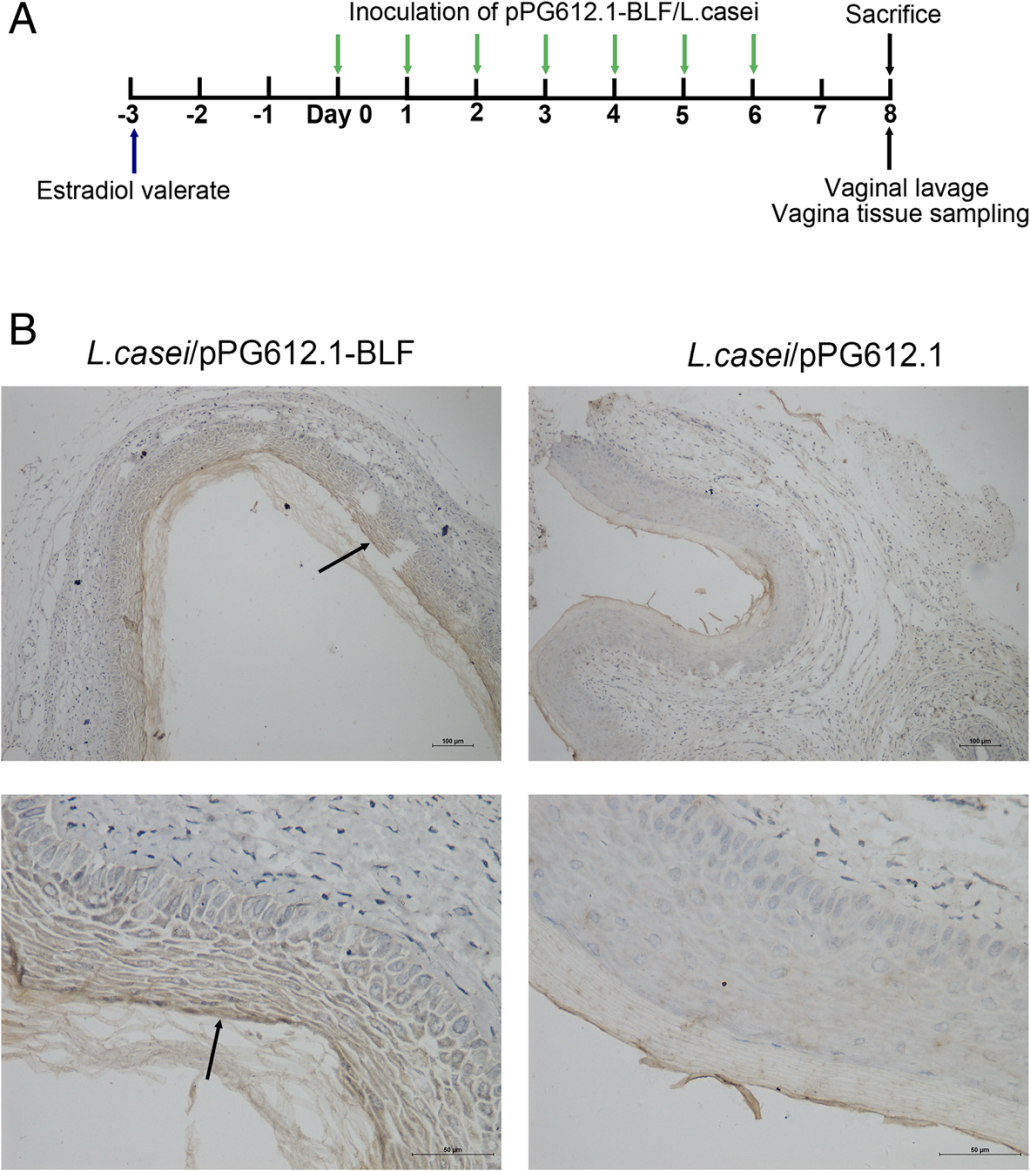
Colonization of murine vagina by *L. casei*/pPG612.1-BLF. **a** Subcutaneous injection of 100 μg of estradiol valerate was given to mice 3 days before inoculation. Twenty microliters of undiluted *L. casei*/pPG612.1-BLF suspension and of the two dilutions were used to inoculate the murine vaginal cavity (5 mice per group) with a pipette at the same time of day for seven consecutive days. Mice were sacrificed by cervical dislocation 2 days after the 7th inoculation; each murine vagina was washed gently with 150 μL of sterile PBS by repeated aspiration, 10 times. The vaginal tissues were fixed in 4% paraformaldehyde and embedded in paraffin prior to immunohistochemical analysis with anti-BLF primary antibodies. **b** A brown positive band (black arrow) was observed above the vaginal epithelial layer from mice which were inoculated with *L. casei*/pPG612.1-BLF into the vagina, while it was not detected in the samples from mice receiving inoculation of pPG612.1/*L.casei*

All animals during the experiment stayed alive before sacrifice. Two days after the inoculation of murine vagina with 20 μL of an undiluted PBS suspension of *L. casei*/pPG612.1-BLF (Fig. 3a), the secreted BLF was detected above the epithelial layer using immunohistochemistry (Fig. 3b). To explore the preventative effects of the *L. casei*/pPG612.1-BLF against VVC in a mouse model, undiluted suspensions of *L. casei*/pPG612.1-BLF, *L. casei*/pPG612.1, and *L. casei* were tested, with 20 μL of PBS as a blank control.

**Fig. 4:**
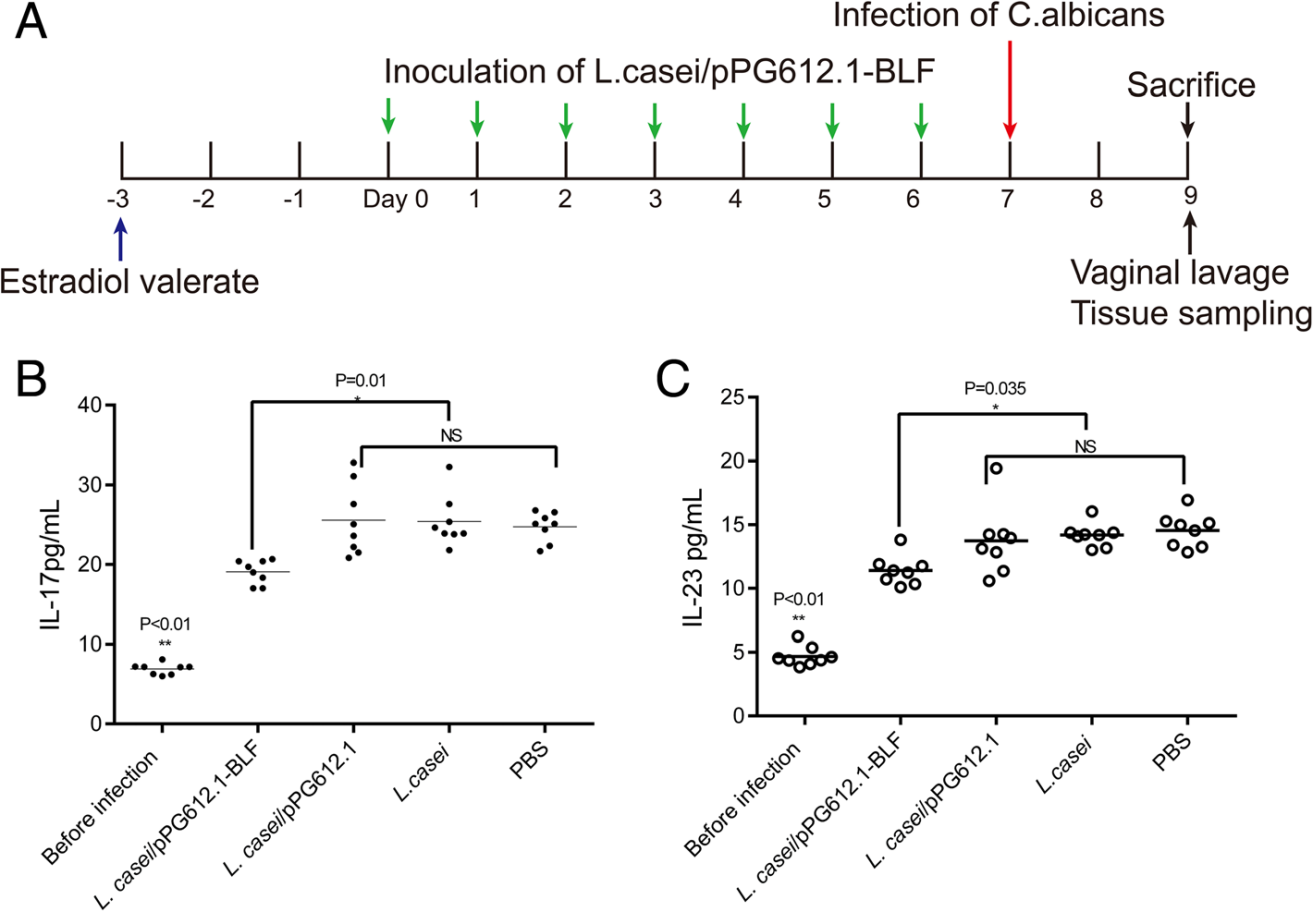
Protective effect of *L. casei*/pPG612.1-BLF in the VVC mouse model. **a** Estradiol valerate was given 3 days before inoculation. Following the application of the *L. casei*/pPG612.1-BLF into the murine vagina, the animals were infected with 20 μL of a *C. albicans* suspension in PBS at 10^8^ CFU/mL delivered into the vaginal cavity. Two days after the infection, all mice were sacrificed and their vaginas were washed with 150 μL sterile PBS prior to being excised and fixed. **b** Vaginal lavages were centrifuged and the supernatant was analyzed for the concentration of IL-17 and IL-23 in the lavage before and after infection by ELISA tests according to the manufacturer’s recommendations. The concentration of IL-17 and IL-23 had been increased after infection with VVC in all mice groups (*P* < 0.01). The concentration of IL-17 was 19.07 ± 1.50 pg/mL in *L.casei*/pPG612.1-BLF group, significantly lower than (*P* = 0.01) those in *L.casei*/pPG612.1 group (25.98 ± 5.10 pg/mL), *L.casei* group (25.42 ± 3.22 pg/mL) and PBS group (24.74 ± 1.87 pg/mL). **c** As for the IL-23, it was lower in *L.casei*/pPG612.1-BLF group (11.43 ± 1.17 pg/mL) (*P* = 0.035), in comparison with that in *L.casei*/pPG612.1 group (13.73 ± 2.67 pg/mL), *L.casei* group (14.20 ± 0.92 pg/mL) and PBS group (14.56 ± 1.35 pg/mL)

Following a 7-day inoculation of the murine vagina with different *L. casei* strains, 20 μL of a *C. albicans* suspension in PBS (10^8^ CFU/mL) was used to inoculate the mouse vagina on day 8 (Fig. 4a). The vaginal lavage of each mouse was collected on day 10, and *C. albicans* CFUs were determined in each sample to analyze the infection burdens. The infection burden in mice receiving *L. casei*/pPG612.1-BLF (4.404 ± 0.040 log_10_CFU/mL) was significantly lower (*P* < 0.01) than in mice receiving *L. casei*/pPG612.1 (4.643 ± 0.035 log_10_CFU/mL), *L. casei* (4.633 ± 0.032 log_10_CFU/mL), and PBS (4.585 ± 0.112 log_10_CFU/mL), indicating that the prophylactic vaginal inoculation with the *L. casei*/pPG612.1-BLF exerted a protective effect against *C. albicans* (Table 1). However, the fungal infection burden in mice receiving *L. casei* strains that did not produce BLF was the same as in PBS group. Similar to the fungicidal effect observed with the two-layer dish assay in vitro, BLF secreted in the murine vagina by the *L. casei*/pPG612.1-BLF strain was able to prevent the invasion and inhibit the growth of *C. albicans* in vivo.

**Table 1:** Infection burden of VVC in groups receiving different treatments pior-infection

Treatment	Infection burden of VVC (log_10_ CFU) post-inoculation
PBS	4.585 ± 0.112
*L.casei*/pPG612.1-BLF	4.404 ± 0.040^*^
*L.casei*/pPG612.1	4.643 ± 0.035
*L.casei*	4.633 ± 0.032

*P < 0.01 in comparison with PBS, L.casei/pPG612.1-BLF and L.casei groups

*C. albicans* stimulates the host’s immune response during infection, including the activation of immune cells and the production of several types of cytokines. IL-17, mainly produced by the Th17 subset of CD4+ lymphocytes, plays an important role in the mucocutaneous candidiasis, positively correlating with the infection burden, namely, higher IL-17 levels correspond to a more serious infection burden [30–32]. Additionally, IL-23 is essential for Th17 cell expansion and function, which is associated with the production of IL-17 [33]. Therefore, we quantitatively analyzed the IL-17 and IL-23 levels in the vagina lavage samples 2 days after the infection. The IL-17 and IL-23 levels increased after *C. albicans* infection in all mouse groups (*P* < 0.01); however, the IL-17 levels were 19.07 ± 1.50 pg/mL in the *L. casei*/pPG612.1-BLF group. They were distinctly lower (*P* = 0.01) than in the *L. casei*/pPG612.1 (25.98 ± 5.10 pg/mL), *L. casei* (25.42 ± 3.22 pg/mL), and PBS groups (24.74 ± 1.87 pg/mL), illustrating a milder VVC infection burden in mice that received a prophylactic inoculation with the *L. casei*/pPG612.1-BLF (Fig. 4b). Similarly, changes in the IL-23 levels were observed; the levels were lower in the *L. casei*/pPG612.1-BLF group (11.43 ± 1.17 pg/mL) (*P* = 0.035) than in the *L. casei*/pPG612.1 (13.73 ± 2.67 pg/mL), *L. casei* (14.20 ± 0.92 pg/mL), and PBS groups (14.56 ± 1.35 pg/mL) (Fig. 4c). Thus, the observed IL-17 and IL-23 levels reconfirmed the notion that the *L. casei*/pPG612.1-BLF strain producing BLF in the murine vagina exhibits a protective role against VVC in vivo. A week after the infection, the percentage of CD4^+^ T cells in the lumber lymph nodes changed because of the activation of the adaptive immune system by *C. albicans* in the vagina. Thus, less CD4^+^ T cells were observed in the *L. casei*/pPG612.1-BLF group than in the control groups (*L. casei*/pPG612.1, *L. casei*, and PBS groups) because less pathogen cells remained in vaginal cavity after being killed by the concentrated BLF secreted by *L. casei*/pPG612.1-BLF.

### Therapeutic effects of *L. casei*/pPG612.1-BLF in the murine VVC model

**Fig. 5:**
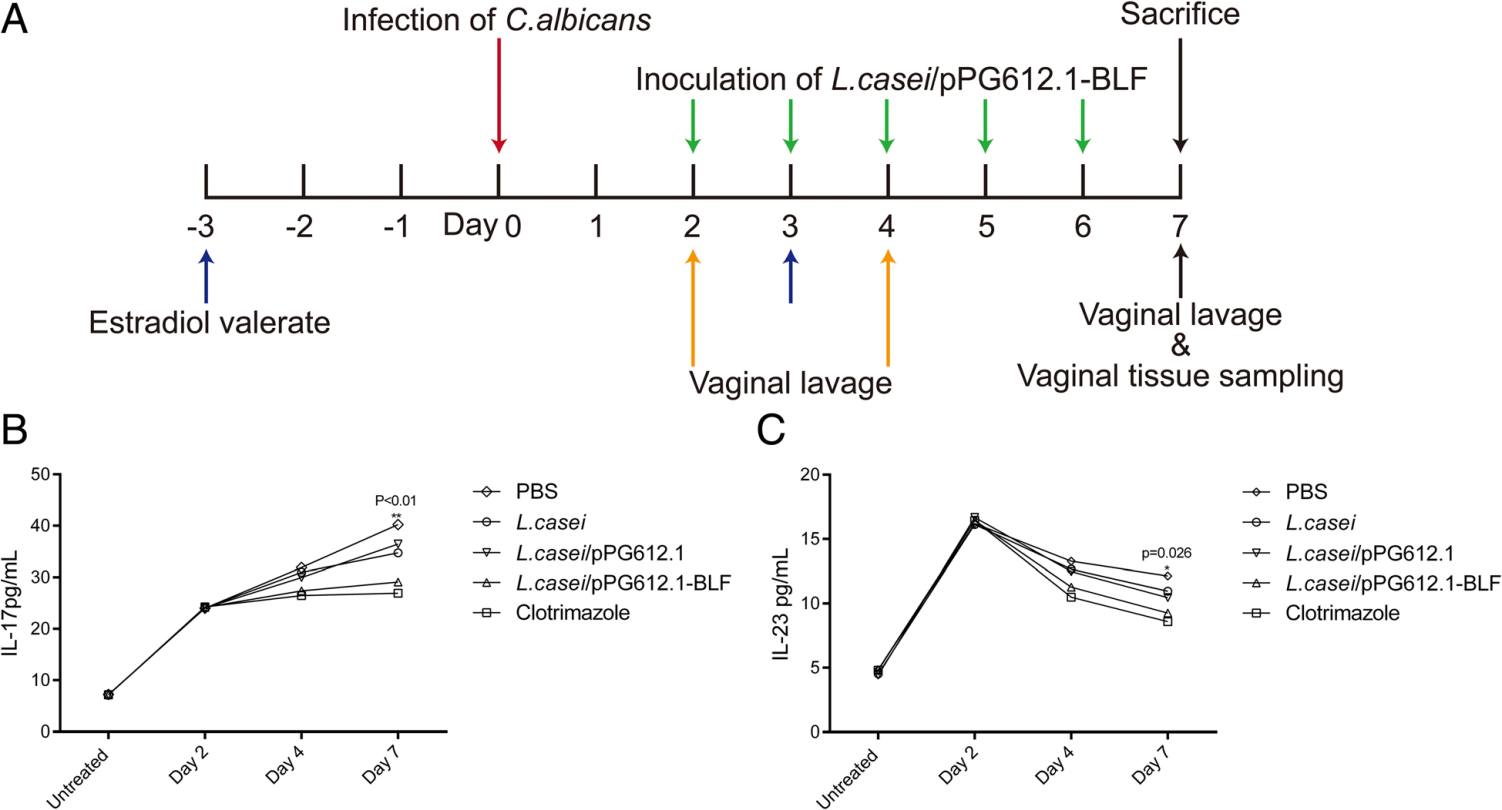
Therapeutic effects of *L. casei*/pPG612.1-BLF in the murine VVC model. **a** 3 days before infection, each mouse received estradiol valerate and this was repeated every 7 days during the experiment. Four days later, the mice were infected with 20 μL of a *C. albicans* suspension (10^8^ CFU/mL) delivered into the vagina. Two days after infection, mice underwent vaginal inoculation with 20 μL of *L. casei*/pPG612.1-BLF (OD_600_ = 0.6–0.7) for five consecutive days, and were sacrificed with on Day 7. Vaginal lavage was collected on Day 2, Day4 and Day7, and vagina and lumbar lymph nodes were excised for cytological and histological tests. **b** The concentrations of IL-17 in all groups were persistently increased after infection during the treatment, but it was the lowest in clotrimazole group and highest in PBS group. After the completion of treatment, the concentration of IL-17 was lower in *L.casei*/pPG612.1-BLF group (29.08 ± 1.33 pg/mL) than that in *L.casei*/pPG612.1 group (36.42 ± 2.80 pg/mL) and *L.casei* group (34.71 ± 2.18 pg/mL). **c** As for IL-23, it was the highest in PBS group (12.15 ± 1.89 pg/mL), while lowest in 20 mg/mL clotrimazole group (8.60 ± 1.02 pg/mL) after the treatment. Importantly, it was significantly lower (*P* = 0.026) in the *L.casei*/pPG612.1-BLF group (9.65 ± 1.74 pg/mL) than the *L.casei*/pPG612.1 (10.46 ± 0.73 pg/mL) and *L.casei* (10.95 ± 1.02 pg/mL) groups

To evaluate the therapeutic effect of the *L. casei*/pPG612.1-BLF strain in the murine VVC, 20 μL of an *L. casei*/pPG612.1-BLF suspension or other agents were delivered into the murine vagina with a pipette for five consecutive days (Fig. 5a). Overall, the infection burdens in all groups were reduced after a 5-day treatment, which was partly attributed to the natural immune response, but the reductions were more remarkable in groups receiving *L. casei*/pPG612.1-BLF or clotrimazole than in the PBS group. Specifically, the infection burden in the *L. casei*/pPG612.1-BLF group was 1.710 ± 0.390 log_10_CFU/mL; this was lower than in the clotrimazole group (0.492 ± 0.170 log_10_CFU/mL) (*P* < 0.001), but higher than that in the *L. casei*/pPG612.1 (3.170 ± 0.252 log_10_CFU/mL) and *L. casei* groups (2.936 ± 0.259 log_10_CFU/mL) (*P* < 0.01) (Table 2).

**Table 2:** Infection burdens of groups receiving different treatments post-infection

Treatment	Infection burden of VVC (log_10_ CFU)	
	Before treatment	After treatment
PBS	4.76 ± 0.341	3.738 ± 0.231
Clotrimazole	4.766 ± 0.284	0.492 ± 0.170^a*^
*L.casei*/pPG612.1-BLF	4.76 ± 0.310	1.710 ± 0.390^b*^
*L.casei*/pPG612.1	4.782 ± 0.279	3.170 ± 0.252^c^
*L.casei*	4.746 ± 0.333	2.936 ± 0.259

^a*^P < 0.01 in comparison with *L.casei*/pPG612.1-BLF

^b*^P < 0.01 in comparison with *L.casei*/pPG612.1, *L.casei* and PBS

^c^*p* > 0.05 in comparison with *L.casei*

Quantitative analysis of the IL-17 levels in the vaginal lavage revealed that the cytokine levels in all groups were persistently elevated during the treatment after the infection; however, this effect was most apparent in mice receiving PBS and least apparent in mice treated with 20 mg/mL clotrimazole. Compared with the *L. casei*/pPG612.1 group (36.42 ± 2.80 pg/mL) and *L. casei* group (34.71 ± 2.18 pg/mL), less IL-17 (P < 0.01) was detected in the *L. casei*/pPG612.1-BLF group (29.08 ± 1.33 pg/mL) after the completion of treatment (Fig. 5b). Regarding the IL-23 levels, they were highest in the PBS group (12.15 ± 1.89 pg/mL) and lowest in the clotrimazole group (8.60 ± 1.02 pg/mL) after the treatment. Moreover, they were significantly lower (*P* = 0.026) in the *L. casei*/pPG612.1-BLF group (9.65 ± 1.74 pg/mL) than in the *L. casei*/pPG612.1 (10.46 ± 0.73 pg/mL) and *L. casei* groups (10.95 ± 1.02 pg/mL) (Fig. 5c).

**Fig. 6:**
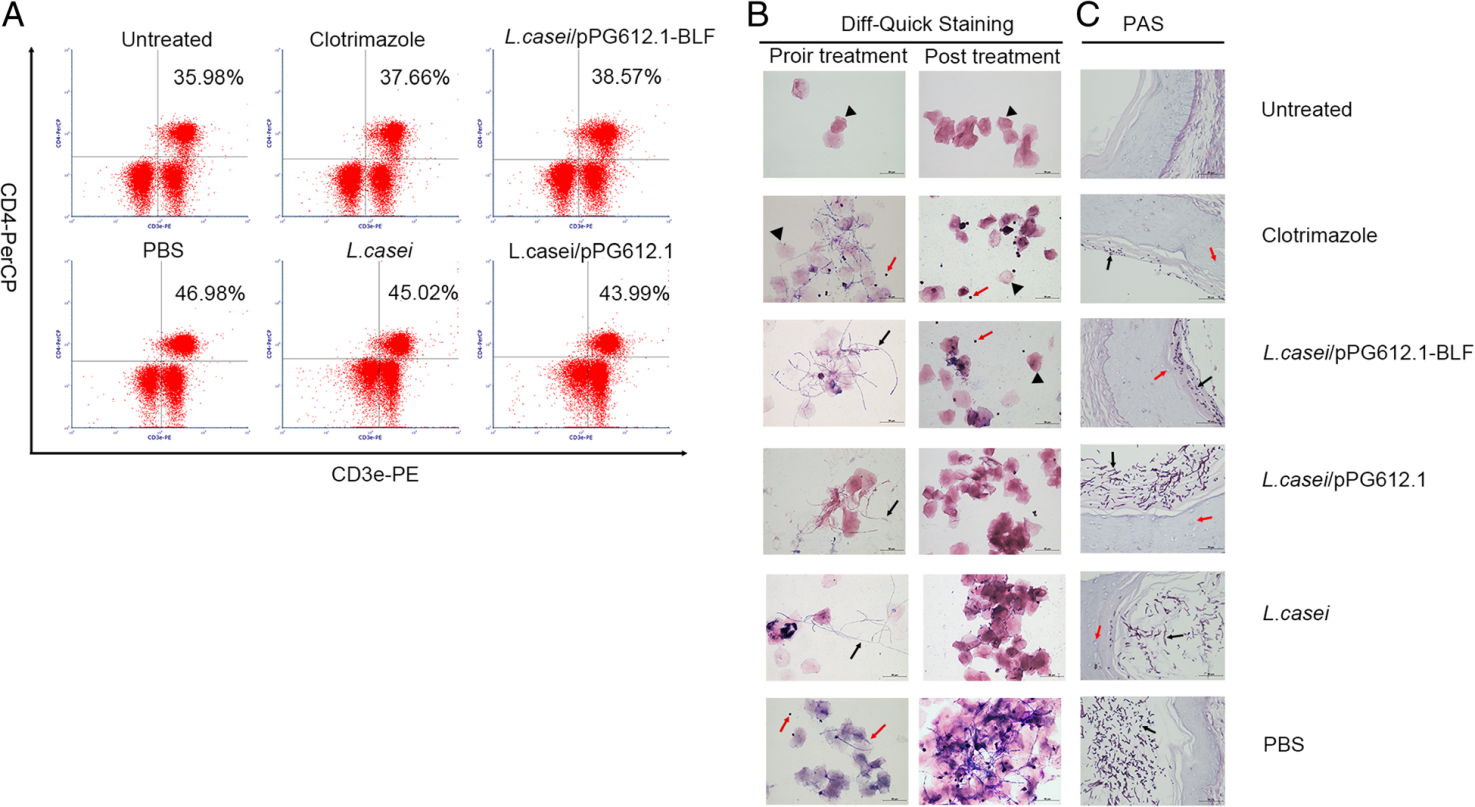
Cytological analysis of lumbar lymph nodes and histological analysis of vagina tissue in murine VVC model after treatment with *L.casei*/pPG612.1-BLF. **a** The change in the percentage of CD4+ T cells in murine lymph nodes were evaluated by dual-colour flow cytometric analysis. Lumbar lymph nodes were isolated immediately after sacrifice to make single-cell suspension at 10^6^cell/mL. Phycoerythrin (PE) -labelled anti-mouse CD3 and peridinin Chlorophyll Protein Complex (PerCP)-labelled anti-mouse CD4 antibodies simultaneously were added to the samples at a concentration of 0.25 μg/10^6^ cells. In the PBS group, the percentage of CD4+ T cells in the murine lumbar lymph nodes was 48.09 ± 3.53%; it was 46.93 ± 2.42% in *L. casei* group and 45.55 ± 2.07% in the *L. casei*/pPG612.1 group, and was higher (*P* = 0.04) than in the *L. casei*/pPG612.1-BLF group (39.34 ± 1.34%) and clotrimazole group (38.65 ± 1.94%). **b** Vaginal cast-off cells in lavage was processed with Diff-Quick Staining prior and post treatment in murine VVC model. Heat-fixed lavage smears were dip into the StainIfor 15 s, and successively into Stain II for another 15 s, followed by rinsed rapidly in tap water. Post-infection with fungi (prior treatment), many filamentous pseudohyphae (violet) and cornified squamous epithelial cells (pink) were apparent in each sample. Post 5-day treatment, fewer epithelial cells were apparent in samples from mice which were given *L.casei*/pPG612.1-BLF or 20 mg/mL clotrimazole, with several neutrophils (red arrow) and no filamentous pseudohyphae. In contrast, in samples from mice receiving *L.casei*/pPG612.1 or *L.casei* or PBS, copious numbers of epithelial cells were observed with filamentous pseudohyphae (PBS group) or without filamentous pseudohyphae (*L. casei*/pPG612.1 and *L. casei* groups). **c** The vagina tissue was extracted following sacrifice post 5-day treatment, fixed in 10% neutral-buffered formalin and embedded in paraffin. The 3-to 4-μm–thick sections from every mouse were mounted on a poly-l-lysine–coated slide and stained with hematoxylin, eosin and periodic-acid/Schiff’s reagent. Much fewer pseudohyphae (black arrow) and blastoconidia adhering to the cornified epithelial layer were observed in samples from mice which were given treatment with *L.casei*/pPG612.1-BLF or 20 mg/mL clotrimazole, in comparison with that from groups which received *L.casei*/pPG612.1, *L.casei* or PBS. Additionally, samples from *L.casei*/pPG612.1 and *L.casei* groups showed fewer pseudohyphae and blastoconidia in the vaginal cavity than those in the PBS group. Besides, infiltration of neutrophil (red arrow) into the mucosal layer were observed in infected mice. Scale bars, 50 μm (**b**), 50 μm (**c**)

As revealed by the dual-color flow cytometry analysis, the change in the percentage of CD4+ T cells in the vaginal draining lymph nodes between pre-infection and post-infection varied among groups. Overall, the fungus triggered the cell-mediated immunity in the local vaginal tissues and the draining lymph nodes, resulting in increased percentage of CD4+ T cells in the lumbar lymph nodes post-infection. Specifically, in the PBS group, the percentage of CD4+ T cells in the murine lumbar lymph nodes was 48.09 ± 3.53%; it was 46.93 ± 2.42% in *L. casei* group and 45.55 ± 2.07% in the *L. casei*/pPG612.1 group, and was higher (*P* = 0.04) than in the *L. casei*/pPG612.1-BLF group (39.34 ± 1.34%) and clotrimazole group (38.65 ± 1.94%). This indicated an effective treatment in the latter two groups, which were able to clear more pathogens, thus reducing the intensity of the immune response in the local vaginal tissues (Fig. 6a).

Vaginal lavage smears from mice with VVC before and after treatment were stained with the Diff-Quik stain. Only few anucleated cornified epithelial cells were observed in samples from healthy untreated mice. Post-infection with fungi (prior treatment), many filamentous pseudohyphae (violet) and cornified squamous epithelial cells (pink) were apparent in each sample. Following a 5-d treatment, in samples from the same mice receiving a vaginal topical treatment with *L. casei*/pPG612.1-BLF or clotrimazole, fewer epithelial cells were apparent than that prior the treatment, with several neutrophils (blue) and no filamentous pseudohyphae (Fig. 6b). In contrast, in samples from mice receiving *L. casei*/pPG612.1, *L. casei*, or PBS, copious numbers of epithelial cells were observed with filamentous pseudohyphae (PBS group) or without filamentous pseudohyphae (*L. casei*/pPG612.1 and *L. casei* groups).

To observe the fungi in the vagina, the tissues were fixed and processed for PAS staining. Unlike in the healthy untreated mice, with no *C. albicans* visible in the vaginal cavity after H&E/PAS staining, the vaginal cavity from infected animals was filled with large numbers of filamentous pseudohyphae and blastoconidia; however, their quantity varied in different groups. Specifically, after treatment with *L. casei*/pPG612.1-BLF or 20 mg/mL clotrimazole, a lot less pseudohyphae and blastoconidia adhering to the cornified epithelial layer were observed than in groups that received *L. casei*/pPG612.1, *L. casei*, or PBS, in agreement with the Vaginal lavage CFU data (Fig. 6c). Moreover, in samples from the *L. casei*/pPG612.1 and *L. casei* groups, fewer pseudohyphae and blastoconidia were seen in the vaginal cavity than in samples from the PBS group. These observations were in agreement with the CFU data, indicating the effectiveness of *L. casei*/pPG612.1-BLF or 20 mg/mL clotrimazole in treating VVC in a murine model.

## Discussion

The yeast form of *C. albicans* is a commensal organism that dwells in the lower genital tract of a healthy female; however, the fungus undergoes a pathogenic transformation when the balance between the pathogen and the host’s immune system is perturbed. During this transformation, a rapid alteration of gene expression occurs such as *SAP, ECE1* and *WOR1* gene [34–36], in response to various environmental stimuli.

The defense of vaginal tissues against the invasion of *Candida* species depends on both innate and adaptive immunity [37, 38], and comprises secreted cytokines, antibodies, AMPs, as well as probiotics – *Lactobacillus* species. All these factors constitute the front-line defense barrier against *Candida* invasion, disturbing the fungal growth, depriving the fungus of nutrients, or regulating the immune response. The maintenance of health in the female vagina is partly attributed to the *Lactobacillus* species. Lactobacilli are dominant probiotic microorganisms; they produce lactic acid and other substances, contributing to the maintenance of a low pH in the vagina, thus preventing the overgrowth of pathogens, especially those causing bacterial vaginosis (BV), gonorrhea, and VVC. Therefore, preservation of the vaginal microbiome and reinforcement of the immunological microenvironment are feasible approaches to protection against infection with *Candida sp*. In the current study, we constructed a *L. casei*/pPG612.1-BLF strain that secreted an AMP, BLF, and tested its efficacy in the defense against and clearing of *C. albicans* in a murine model of VVC.

Human-derived lactoferrin (hLF) is already produced in *L. casei* and was shown to exert antibacterial activity in the murine gastrointestinal tract [39]. The antifungal activity of lactoferrin (human-derived and bovine-derived) stems from its ability to sequester iron, effectively starving *Candida* species for iron, and leading to membrane damage and leakage via iron-independent ways [23]. The plasmid pPG612.1, a mature plasmid secretion vector for *Lactobacillus* species, contains the ssUsp secretion signal system, and has already been successfully used for heterologous protein production in *L. casei* [40, 41]. In the two-layer agar dish assay in the current study, BLF that was constitutively produced by *L. casei* diffused into the SDA layer, killing *C. albicans* cells and suppressing the growth of survivors. Despite not being 100% effective, the same antifungal effect was also observed in the mouse model, and the fungal infection burden during VVC was apparently lower in mice that have been prophylactically inoculated with *L. casei*/pPG612.1-BLF before fungal infection than in mice inoculated with the non-BLF–secreting strains. The exogenous BLF was secreted by *L. casei*/pPG612.1-BLF into the extracellular environment, participating in the defense against the invasion of *C. albicans*. In contrast, the non-BLF–secreting *L. casei* strains were not effective in defending the vaginal mucosa against *C. albicans*. Moreover, the combined results of the histological analysis of vaginal tissues, changes in the infection burdens (judged by the vaginal lavage analysis), and cytological analysis of the lumbar lymph nodes all revealed the pronounced ability of *L. casei*/pPG612.1-BLF to clear the *C. albicans* cells in the murine model of VVC.

The immunity mediated by T helper 1 (Th1) and T helper 17 (Th17) cells, the subsets of CD4+ T cells, is considered to be the main host defense mechanism during the clearance of *C. albicans* colonizing the vagina. These cells secrete diverse cytokines, e.g., IL-17 and IL-22, engaging in the host-pathogen interactions [42]. The protective role of IL-17, which is mainly secreted by T cells after activation by IL-23, is well recognized. The Th17/IL-17 pathway is involved in the secretion of AMPs and proinflammatory cytokines, as well as neutrophil recruitment [30]. Particularly, the IL-17 and IL-23 dynamics are different during VVC, with increased IL-17 levels and reduced IL-23 levels after an infection, and the IL-17 levels are positively correlated with the VVC infection burden [43]. This was also observed in the current study, with the IL-17 levels lower in the *L. casei*/pPG612.1-BLF group than in *L. casei*/pPG612.1, *L. casei* and PBS groups. *Lactobacillus casei*/pPG612.1-BLF worked synergistically with the host immune system to defend the host against *C. albicans* invasion or clear the colonizing *C. albicans* cells, resulting in lower pathogen counts in the vagina and lightening the immunity activity; his was verified by the reduced IL-17 and IL-23 levels in the vaginal lavage and the percentage of CD4+ T cells in the lumbar lymph nodes. Two days post-infection, a massive influx of neutrophils, the effector cells like macrophage and neutrophil of Th17 lineage through IL-17, infiltrated the vaginal cavity to mount a protective response against candidiasis [44]. After the *L. casei*/pPG612.1-BLF treatment, the amount of vaginal discharge, including neutrophils and cast-off cornified squamous epithelial cells, was reduced, indicating remission of the VVC.

## Conclusion

Overall, as a prophylactic agent, *L. casei*/pPG612.1-BLF acted as an enhancer, improving the immunity of vaginal mucosa against the intrusion of *C. albicans*. Furthermore, the strain efficiently reduced the fungal burden in VVC after a 5-d treatment, although the therapeutic effect was inferior to 20 mg/mL clotrimazole. This might be improved by optimizing the dosage of the *L. casei*/pPG612.1-BLF or the therapeutic schedule. Although additional pathogenic *Candida* species and non-*Candida* species should be tested in further studies before making definitive conclusions, considering the unparalleled advantages of the *L. casei* strain, i.e., production of a natural AMP and its probiotic nature, the *L. casei*/pPG612.1-BLF might be a promising agent for treating VVC in clinical practice.

## Methods

### Bacteria and culture medium

*Lactobacillus casei* ATCC 393 (American Type Culture Collection, Manassas, Vaginia, USA) was cultured anaerobically in the Mann Rogosa Sharpe (MRS) broth (Biosharp, Hefei, China) at 37 °C without shaking. Transformed *L. casei* cells were plated on the MRS agar containing 10 μg/mL chloromycetin (Cm) and cultured in a sealed container at 37 °C. For protein production, *L. casei* was cultured in MRS containing 2% (*w*/*v*) xylose instead of sucrose. *Escherichia coli* DH5α CCTCC AB 2012883 (China Center for Type Culture Collection, Wuhan, China) was grown in LB (Biosharp, Hefei, China) medium at 37 °C with 100 μg/mL ampicillin, when harboring the plasmid pUC57 (Sangon Biotech Shanghai Co., Shanghai, China), or with 30 μg/mL Cm, when harboring pPG612.1 (see below). *C. albicans* ATCC 10231 (American Type Culture Collection) was grown in Sabouraud dextrose broth (SDB; Biosharp) at 37 °C with shaking at 180 rpm.

### Expression plasmid construction

The pPG612.1 plasmid (BioVector NTCC Inc., Beijing, China) is an expression vector derived from the lactococcal plasmid pWV01, with the ability to replicate in *E. coli* and Lactobacillus sp. [41]. was purchased from BioVector NTCC Inc., Beijing, China. BLF gene fragment (NP_851341.1) with BamHI site at the 5′-terminus and XhoI site at the 3′-terminus was synthesized (Synbio Technologies, Suzhou, China) and cloned into the plasmid pUC57, resulting in pUC57-BLF. Plasmid pUC57-BLF was amplified in *E. coli* DH5α and purified using the E.Z.N.A plasmid mini kit (Omega Bio-tek, Inc., Norcross, GA, USA). The BLF fragment was retrieved from the pUC57-BLF using BamHI and XhoI enzymes (New England Biolabs Inc., Ipswich, MA, USA) and ligated into the corresponding sites of pPG612.1, generating the plasmid pPG612.1-BLF. The ligation reaction mix was used to transform *E. coli* DH5α and the resulting transformants were confirmed by PCR amplification and sequencing (Table 3).

**Table 3:** Bacterial strains, plasmids, and primers used in the current study

Material	Description	Reference or source
Bacteria		
*L. casei*	Plasmid free	ATCC393
*E. coli DH5α*	Transformation host	CCTCC AB 2012883
*L. casei*/pPG6121.-BLF	Secreted BLF producer	This work
*L. casei*/pPG6121	Transformation host	This work
Plasmid		
pUC57	Amp^r^, ori	Sangon (China)
pPG612.1	Amp^r^, Cm^r^, ori	Li et al.
pPG612.1-BLF	Amp^r^, Cm^r^, ori	This work
Primers		
BamHI-F	TGGATCTTGCTTTGAAGGGT	This work
BamHI-R	TTTCGTCCCATAGATCTCTGC	This work
XhoI-F	ACACGTGAAACAGGTGCTGCT	This work
XhoI-R	TTCGTCCAACCAAACCGACT	This work

*ATCC* American Type Culture Collection, *CCTCC* China Center for Type Culture Collection

Plasmid pPG612.1-BLF was electroporated into *L. casei* according to a standard protocol [45]. Briefly, 1 μg of pPG612.1-BLF was added to 200 μL of *L. casei* (OD600 = 0.6), gently mixed, and kept on ice for 10 min. The mix was transferred into an ice-cold electroporation cuvette with a 2-mm electrode gap, and subjected to a single electric pulse (1.5 kV, 25 μF, and 400 Ω.) using a Bio-Rad GenePulser (Bio-Rad Laboratories, Inc., Berkeley, CA, USA). The mix was diluted in 1 mL of fresh MRS broth and incubated anaerobically at 37 °C for 1 h before spreading on MRS agar containing 10 μg/mL Cm. Plasmid pPG612.1-BLF was isolated from the L. casei/pPG612.1-BLF strain using the E.Z.N.A plasmid mini kit and were identified by PCR and sequencing via ABI PRISM 3130 sequencer (Applied Biosystems, Foster City, CA, USA).

### Protein expression and Western blotting

The strain *L. casei*/pPG612.1-BLF was cultivated in 200 mL of MRS broth supplemented with 2% xylose. An overnight culture was centrifuged at 8000×*g* for 3 min, and the pellet was washed twice in PBS prior to resuspending in the lysis buffer (50 mM Tris-HCl, pH 8.0, 10% glycerol, 0.1% Triton-X-100, 100 μg/mL lysozyme, and 1× proteinase inhibitor cocktail). Following sonication (21% output for 3-s pulse and 4-s rest, for 12 min) in an SCIENTZ-IID apparatus (Scientz, Zhejiang, China), the sample was centrifuged at 10,000×*g* for 50 min, and the supernatant was analyzed by western blotting. In addition, protein from the supernatant of the overnight culture was precipitated using 10% trichloroacetic acid, and resuspended in pure water, before analysis by western blotting.

The proteins were transferring onto a PVDF membrane, and the membrane was blocked in a blocking solution (P0252, Beyotime, Shanghai, China) for 1 h at 25 °C. The blots were washed six times in Tris-buffered saline with 0.05% Tween-20 for 30 min and between all subsequent steps for 5 min. The blots were incubated with monoclonal mouse anti-BLF primary antibodies at the ratio of 1:1000 (ab10110, Abcam, Cambridge, UK) overnight at 4 °C, and then with a horseradish peroxidase (HRP)-conjugated goat anti-mouse IgG (A0216, Beyotime). Finally, the blots were incubated with the chemiluminescent HRP substrate (ECL, Millipore, Billerica, Massachusetts, USA) and the immunolabeled bands were visualized according to the manufacturer’s instructions.

### Fungicidal activity of *L. casei*/pPG612.1-BLF in vitro

To evaluate the fungicidal effect of *L. casei*/pPG612.1-BLF, a two-layer agar dish assay was developed, that provided a commensal-like environment for *L. casei* and *C. albicans*, allowing a direct exposure of *C. albicans* to *L. casei* secretions. Sterile MRS agar and Sabouraud dextrose agar (SDA), both containing 1.5% low-melting agarose, were kept in a water bath at 37 °C. Before the experiment, 20 μL of *L. casei*/pPG612.1-BLF (OD600 = 0.6) was added to 200 mL of MRS medium and mixed gently to avoid bubbles, in the water bath set at 37 °C. Before cooling down, 20 mL of the mix was poured onto a 60-mm diameter culture dish and allowed to solidify. For a two-layer agar dish, 10 mL of SDA was poured on top of the MRS agar, and then allowed to solidify. Following that, 100 μL of *C. albicans* suspension containing ca. 100 cells was placed on the surface of the two-layer agar dish, i.e., the SDA surface. Two-layer agar dishes containing *L. casei*/pPG612.1, *L. casei*, or no *L. casei* were negative controls; one-layer SDA dishes (without MRS) containing *C. albicans* alone were blank controls. All dishes were incubated in an inverted position at 37 °C for 48 h, and their photographs were taken every 12 h. The fungicidal activity of *L. casei*/pPG612.1 was assessed based on the number and average size of *C. albicans* colonies in each group. The experiment was repeated on two independent occasions with triplicate in each group.

### Experimental animals and compliance with ethical standards

A total of 150 healthy female Balb/c (H-2d) mice (6–8-week-old, 16–18 g) were obtained from Dossy Experimental Animal Ltd. (Chengdu, China) used in the current study. Five mice were housed per one cage, and the animals were supplied with food and water ad libitum. The care, maintenance, and handling of the animals followed the requirements of the NIH guidelines for experiments with animals [46]. The animal experiment was approved by the Ethics Committee of West China Second Hospital of Sichuan University. All animal experiments were performed in duplicate.

### Colonization of murine vagina by L. casei/pPG612.1-BLF

The strain *L. casei*/pPG612.1-BLF grown in 1 mL MRS broth containing 2%(*w*/*v*) xylose was harvested at OD_600_ = 0.6–0.7 and adjusted to an approximate concentration of 5 × 10^7^–1 × 10^8^ cells/mL, washed three times with 1 mL sterile PBS, and resuspended in 1 mL fresh PBS. One hundred microliter of the suspension was diluted in PBS at the ratio of 1:10 and 1:100. Then, 20 μL of undiluted *L. casei*/pPG612.1-BLF suspension and of the two dilutions were inoculated into the murine vaginal cavity (5 mice per group) with a pipette at the same time of day for seven consecutive days, under the inhalation anesthesia with isoflurane (Adamas Reagent, Ltd. Shanghai,China) in a respiratory chamber with the flow rate of oxygen at 2 L/min [47]. The mice were sacrificed by cervical dislocation 2d after the 7th inoculation; each murine vagina was washed gently with 150 μL of sterile PBS by repeated aspiration, 10 times. To verify the colonization of *L. casei*/pPG612.1-BLF, 100 μL of the lavage was plated on MRS agar; 2 μL was also used as a template in a PCR reaction to amplify the plasmid pPG612.1-BLF, using the primers mentioned above. The vaginal tissues were fixed in 4% paraformaldehyde (Biosharp) and embedded in paraffin prior to immunohistochemical analysis with anti-BLF primary antibodies.

### Prophylactic usage of *L. casei*/pPG612.1-BLF against murine VVC

As specified elsewhere [48], 3 days before inoculation of *L. casei*/pPG612.1-BLF, each mouse received subcutaneous injection of 100 μg of estradiol valerate (Aladdin Industrial Co., Shanghai, China) in 100 μL of sesame oil (Bellancom Chemistry Co., Beijing, China); this was repeated every 7 days during the experiment.

Following the application of the *L. casei*/pPG612.1-BLF into the murine vagina, the animals were infected with 20 μL of a *C. albicans* suspension delivered into the vaginal cavity. Briefly, *C. albicans* cells grown in SDB were harvested at OD_600_ = 0.5–0.6, washed three times with sterile PBS, and resuspended in PBS at 10^8^ CFU/mL. Mice already harboring the *L. casei*/pPG612.1-BLF were infected with 20 μL of *C. albicans* suspension into the vaginal cavity (10 mice) under inhalation anesthesia as described above and kept upside-down for 5 min. The same amounts of L. casei/pPG612.1 and *L. casei* were used as negative controls; PBS was used as a blank control (10 mice per group). Two days after the infection, all mice were sacrificed and their vaginas were washed, as described above. Immediately after cervical dislocation, the vaginal tissues and lumbar lymph nodes were excised, and fixed in 4% paraformaldehyde. To assess the *C. albicans* infection burdens in each mouse, 10 μL of the lavage was diluted in 190 μL of PBS, spread on SDA, and incubated at 37 °C for 48 h. The remaining vaginal lavage was centrifuged and stored at − 70 °C until analysis by an enzyme-linked immunosorbent assay (ELISA; see below).

### Therapeutic usage of the *L. casei*/pPG612.1-BLF in a murine model of VVC

Three days after subcutaneous of estradiol valerate, the mice (10 mice per group) were infected with 20 μL of a *C. albicans* suspension (10^8^ CFU/mL) delivered into the vagina, as described above. To assess the infection burden, 2 days after the infection, the murine vagina was washed with 150 μL of PBS; from that day on, the mice underwent vaginal inoculation with 20 μL of *L. casei*/pPG612.1-BLF (OD_600_ = 0.6–0.7) for five consecutive days [49]. The groups given *L. casei*/pPG612.1 or *L. casei* were negative controls; PBS was a blank control; and 20 mg/mL of clotrimazole (Q.d.) (Tokyo Chemical Industry Co., Tokyo, Japan) was a positive control. On day 3 of the treatment, each mouse vagina was washed as described above under inhalation anesthesia with isoflurane (Adamas Reagent, Ltd). Upon sacrifice on day 5 of treatment, the murine vagina was washed with 150 μL of PBS, and the vaginal tissue was excised and fixed in 4% paraformaldehyde. The lumbar lymph nodes were excised and immediately prepared for dual-color flow cytometry analysis (see below). All vaginal lavage samples were processed in the same way; 10 μL of the lavage was spread on SDA and *C. albicans* CFUs determined after 48 h incubation at 37 °C; the rest of the sample was kept at − 70 °C before ELISA.

### Analysis of interleukins (ILs) and cast-off cells in the vaginal lavage

The presence of ILs in the vaginal lavage was analyzed using an ELISA kit (SEA063Mu for IL-17, SEA384Mu for IL-23, Cloud-Clone Corp, Wuhan, China), according to the manufacturer’s recommendations. Briefly, 100 μL of the supernatants and reaction standards were placed in a 96-well plate pre-coated with a biotin-conjugated antibody; the solutions were discarded after an incubation at 37 °C for 1 h. Then, 100 μL of a specific horseradish peroxidase-conjugated antibody solution was added to each well, and incubated at 37 °C for 1 h; the wells were then washed three times with PBS containing 1% Tween-20. Next, 90 μL of a tetramethylbenzidine substrate solution was added to each well and incubated at 37 °C for 10 min to allow for the color to develop. Finally, 50 μL of a sulfuric acid solution was added to each well to stop the reaction; the plates were immediately placed in a Model 680 microplate reader (Bio-Rad Laboratories, Inc.), and sample absorbance was measured at 450 nm.

The vaginal cast-off cells were visualized using the Diff-Quick stain (Biosharp). Briefly, heat-fixed lavage smears on glass slides were dipped in stain I for 15 s, then in stain II for 15 s; this was followed by a rapid rinse in tap water. The neutrophils were observed as cells with blue nucleus, pink cytoplasm, and violet granules; the cornified epithelial cells were stained pink; the fungi were stained blue. All slides were examined under a Leica DM1000 microscope (Leica Microsystems, Wetzlar, Germany).

### Cytological analysis of lumbar lymph nodes

Dual-color flow cytometry was used to evaluate the relative changes in the percentage of CD4^+^ T cells in the lumbar lymph nodes after the VVC treatment with *L. casei*/pPG612.1-BLF. The lymph nodes were isolated from the posterior abdominal wall lateral to the abdominal aorta, and transferred into a clean dish containing RPMI-1640 medium (Corning Inc., New York, USA); they were then cut into smaller pieces. The suspension was filtered through a 70-μm cell strainer and the filtrate was centrifuged at 1500×*g* for 15 min. The cell pellets were washed two times in sterile PBS, and suspended in PBS to obtain a single-cell suspension of 10^6^ cells/mL. Phycoerythrin (PE) -labeled anti-mouse CD3 (100,431, Biolegend, San Diego, CA, USA) and peridinin Chlorophyll Protein Complex (PerCP)-labeled anti-mouse CD4 (100,431, Biolegend) antibodies were simultaneously were added to the samples at a concentration of 0.25 μg/10^6^ cells; the mixtures were left to react on ice for 30 min in the dark before analysis. The flow cytometry analysis was performed using a Cytomics FC 500 instrument (Beckman Coulter, Inc., Pasadena, CA, USA), and the data were collected and analyzed using FCS Express 6 (De Novo Software, Glendale, CA, USA).

### Histological analysis of the vaginal tissue

The murine vagina was entirely extracted, and then fixed in 4% paraformaldehyde (48 h at 4 °C) after wash with fresh PBS. The tissues were then embedded in paraffin before being sectioned transversely into 3 μm–thick sections. The tissue sections from each mouse were placed on poly-l-lysine–coated slides and stained with hematoxylin, eosin, and periodic-acid/Schiff’s reagent (PAS) [50]. The slides were observed under a Leica DM1000 microscope (Leica Microsystems, Wetzlar, Germany).

### Statistical analysis

Comparison of the infection burden of VVC in different groups was analyzed using the Mann–Whitney U-test done using SPSS (version 22, IBM, Armonk, NY, USA). The other quantitative data was analyzed by one-way analysis of variance (ANOVA) in SPSS. Significance was set at a *P*-value < 0.05 in all analysis.

## Abbreviations

BLF: Bovine lactoferrin
*C. albicans*: *Candida. Albicans*
*L.casei*: *Lactobacillus casei*
MRS: Mann Rogosa Sharpe
PBS: Phosphate-buffered saline
SDA: Sabouraud dextrose agar
VVC: Vulvovaginal candidiasis
